# Acute Hydrocephalus due to Secondary Leptomeningeal Dissemination of an Anaplastic Oligodendroglioma

**DOI:** 10.1155/2009/370901

**Published:** 2009-12-20

**Authors:** Andreas M. Stark, Heinz-Herrmann Hugo, H. Maximilian Mehdorn, Friederike Knerlich-Lukoschus

**Affiliations:** Department of Neurosurgery, Schleswig-Holstein University Medical Center, Campus Kiel, Arnold-Heller-Str. 3, 24105 Kiel, Germany

## Abstract

Secondary leptomeningeal dissemination of oligodendroglioma is very rare. We report the case of a 38-year-old Caucasian male who presented with acute hydrocephalus. 8 months before, the patient had undergone craniotomy for right frontal anaplastic oligodendroglioma, WHO grade III. By that time, there was no evidence of tumor dissemination. MRI now ruled out local tumor progression but revealed meningeal contrast enhancement along the medulla, the myelon, and the cauda equina. Repeated lumbar puncture revealed increased cerebro-spinal fluid (CSF) pressure and protein content. Malignant cells were not detectable. Surgical treatment consisted in (1) placement of an ommaya reservoir for daily CSF puncture, (2) Spinal dural biopsy confirming leptomeningeal oligodendroglioma metastasis, and (3) ventriculo-peritoneal shunt placement after CSF protein has decreased to 1500–2000 mg/l.

## 1. Introduction

Oligodendroglioma represents about 5% of glial tumors. The clinical course is particularly characterized by seizures. The preferred location is in the cortex and white matter of the cerebral hemispheres, most often located in the frontal lobe. Nevertheless, oligodendroglioma has also been reported in the cerebellum, brain stem, or spinal cord [1].

Median postoperative survival is in the range of 3–5 years. They tend to recur locally. Malignant progression from WHO grade II to III or from II/III to IV (glioblastoma) is not infrequent [1].

The tumor usually presents as a solid mass. However, several cases of primary diffuse leptomeningeal oligodendrogliomatosis have been reported [2–5]. Most of these patients were children or young adults [2, 4, 5]. In contrast, secondary leptomeningeal dissemination from a solid tumor mass is extremely rare [6].

We report the case of a 38-year-old male with secondary cranial and spinal leptomeningeal tumor dissemination 8 months after surgical resection of an anaplastic oligodendroglioma WHO grade III of the right frontal lobe. The treatment was intended to prove the dissemination histologically and to treat hydrocephalus appropriately.

## 2. Case Presentation

### 2.1. History and Examination

**Figure 1:**
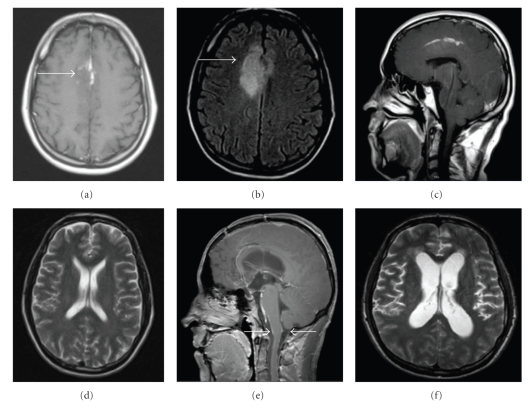
(a)–(d) Cranial MRI at initial presentation before surgical treatment of right frontal anaplastic oligodendroglioma. (a): axial T1-weighted contrast-enhanced MRI, (b) axial fluid-attenuated inversion recovery (FLAIR), and (c) sagittal T1-weighted contrast-enhanced MRI revealed a hyperintense lesion on the right cingulate gyrus with slight local mass effect (arrows). (d): T2-weighted axial MRI showing regular ventricular size. (e)-(f): MRI at second presentation 8 months after initial surgery. (e): sagittal T1-weighted contrast-enhanced MRI showing meningeal enhancement in the craniocervical junction (arrows). (f): Axial T2-weighted MRI showed marked ventricular enlargement.

A 38-year-old patient presented to our department with the 4-day history of headache, nausea, vomiting, and visual disturbance. He also complained about progressive back pain radiating into both legs. The patient had undergone subtotal tumor resection for anaplastic oligodendroglioma (WHO grade III, MIB1 proliferation index 50%) of the right frontal lobe 8 months before. At the time of initial surgery, there were no signs of leptomeningeal tumor involvement or hydrocephalus (Figures 1(a) to 1(d)). Surgery was followed by radiotherapy.

**Figure 2:**
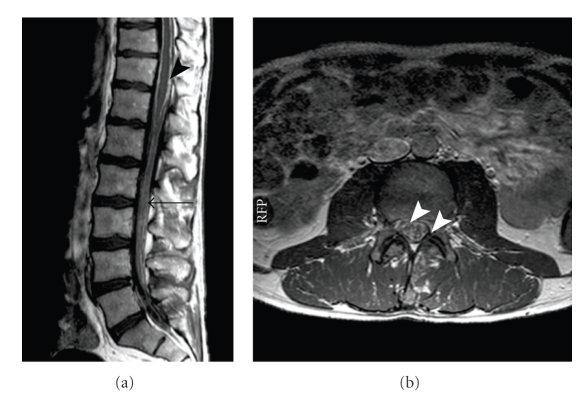
(a) Sagittal and (b) transversal T1-weighted contrast-enhanced magnetic resonance imaging (MRI) of the spine demonstrates meningeal enhancement along the lumbar spinal canal (arrows). Additional enhancement was seen in the cauda equine nerve roots. The biopsy was performed on level L 2/3 (long arrow in (a), level of (b)).

Cranial computed tomography (CCT) on admission now revealed marked ventricular enlargement. Magnetic resonance imaging (MRI) exhibited enlargement of the lateral ventricles and the third ventricle with periventricular high intensity signal on T2WI suggesting transependymal absorption or tumor spreading (Figures 1(e) and 1(f)). There were no signs of local tumor recurrence. Meningeal contrast enhancement was detected around the medulla (arrow head in Figure 1(f)). MRI of the whole spine showed enhancement along the leptomeninges and partially nodular enhancement of the dura. The cauda equina fibers appeared thickened and clotted together (arrow head in Figures 2(a) and 2(b)). Lumbar puncture repeatedly revealed yellow and muddy CSF with an increased opening pressure of about 50 cm H_2_O. CSF examination showed marked elevation of protein (15972 mg/l), slightly elevated cell count (16/*μ*L), and normal glucose and lactate. Cytological CSF examination showed monocytes and lymphocytes but was free of tumor cells.

### 2.2. Surgical Treatment

An Ommaya reservoir was placed for daily CSF puncture. Herein, CSF protein content was decreasing to an amount of 1500–2000 mg/l. Yet there were no tumor cells detectable in consecutive cytological CSF examinations. MRI had shown a marked dural contrast enhancement on the L 2/3 level. So, we decided to biopsy the dura and the nerve fibers on this level. Intraoperatively the nerve fibers were covered with a grayish layer while the dura appeared normal. Biopsies were taken from the nerve fibers and the dura.

**Figure 3:**
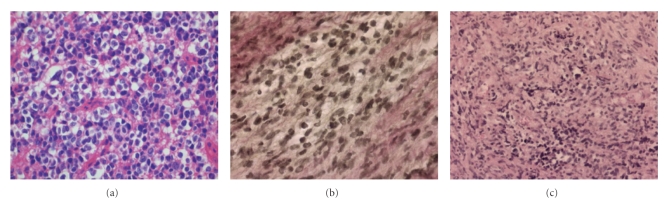
(a) Histopathological image of the primary tumor showing moderate cellularity and a rounded, clear cytoplasm. Histological examination of the dura and the nerve roots revealed infiltration by cells of the anaplastic astrocytoma: (b) Hematoxylin-Eosin staining, magnification 250-fold. (c) Elastica-van-Gieson staining showed tumor cells within dural collagen fibers, magnification 500-fold.

Histological examination confirmed tumor cell infiltration of the dura and nerve fibers by the known anaplastic oligodendroglioma (Figure 3). Remarkably, intraoperatively drawn CSF was again free of tumor cells. Daily puncture of the Ommaya reservoir became insufficient for CSF drainage. So, a ventricular-peritoneal shunt (medium pressure) was inserted after the CSF protein content decreased to an amount of approximately 1500 mg/l.

### 2.3. Postoperative Follow-Up

Postoperatively, PCV chemotherapy (procarbazine, lomustine, vincristine) was planned but refused by the patient. Four months after presentation with hydrocephalus, the patient was clinically stable without any signs of shunt malfunction or tumor progression.

## 3. Discussion

**Primary and Secondary Dissemination of Oligodendroglioma**

Oligodendroglioma mostly arises as a solid mass but can also appear as a diffuse leptomeningeal lesion (oligodendrogliosis or primary diffuse leptomeningeal gliomatosis, PDLG). PDLG is assigned a fatal diagnosis and is frequently associated with hydrocephalus [1–5, 7]. It is assumed that PDLG derives from heterotopic nests of neuroglial tissue in the meninges [8].

In contrast, secondary spinal leptomeningeal dissemination from oligodendroglioma is very rare. In 1977, Arseni et al. reported two cases of secondary spinal involvement in patients with oligodendroglioma from the pre-CT era. The WHO grade is not available. In one case of an 8-year-old boy with pubertas praecox, cachexia, and visual disturbance ventriculography revealed a tumor of the third ventricle. Subtotal tumor resection was followed by radiotherapy. 2 years later, the patient presented with a spinal subdural tumor from C1 to C5 which was again treated surgically. The second patient was a 39-year-old male with the history of left-sided Jackson seizures. Angiography revealed a right temporal lesion which was excised and diagnosed as oligodendroglioma. Radiotherapy was applied. 15 months later, the patient presented with spinal pain and progressive paraparesis. Laminectomy from T1 to T3 revealed a spinal subdural oligodendroglioma [6].

### 3.1. Surgical Treatment of Tumor-Induced Hydrocephalus

Treatment of hydrocephalus in patients with central-nervous system tumors is often a challenge due to the following facts: (A) it remains unclear if raised CSF protein content may cause shunt dysfunction. Most surgeons believe not only that high CSF protein content affects valve function but also that it affects viscosity [9]. According to manufacturers` information, modern shunt valves should tolerate protein content up to 2000–3000 mg/dl. However, there is no threshold the surgeon could rely on. (B) shunt insertion might lead to dissemination of tumor cells into the blood stream or peritoneum. This incidence might be controlled by systemic chemotherapy. (C) tumor dissemination might be difficult to prove [7]. If the CSF is cytologically tumor free, biopsy of gadolinium-enhancing structures identified in MRI might be necessary. Notably, hydrocephalus in patients who underwent surgical intervention might have other causes: (A) Subarachnoid bleeding, either tumor-induced [10] or related to surgery and (B) bacterial meningitis.

In our patient, we had to consider all these facts in order to apply adequate treatment. The raised CSF protein content was brought back to acceptable values by daily puncture of an ommaya reservoir. Since cytological examination of repeated lumbar and reservoir puncture was negative for tumor cells, spinal dural biopsy was performed in order to establish the diagnosis. Finally, ventriculo-peritoneal shunting was performed for persistent hydrocephalus in combination with PCV chemotherapy (which was finally refused by the patient).

## 4. Conclusion

Secondary cranial and spinal dissemination in patients with anaplastic oligodendroglioma is extremely rare. It should be kept in mind when these patients report with signs of hydrocephalus or back pain. The diagnostic method of choice is MRI before and after contrast administration. Cytological CSF examination might be negative. As a consequence, surgical biopsy might be necessary. Treatment of hydrocephalus can include emergency lumbar puncture (after CT or MRI), ommaya reservoir placement, and shunt insertion. The latter should be accompanied by chemotherapy in order to prevent metastasis to other organs.
